# The biomechanical significance of pulley on binocular vision

**DOI:** 10.1186/s12938-016-0280-0

**Published:** 2016-12-28

**Authors:** Hongmei Guo, Zhipeng Gao, Weiyi Chen

**Affiliations:** Shanxi Key Laboratory of Material Strength and Structural Impact, Institute of Applied Mechanics and Biomedical Engineering, College of Mechanics, Taiyuan University of Technology, Yingze West Street 79, Taiyuan, 030024 Shanxi China

**Keywords:** Extraocular muscles, Equilibrium, Force, Modeling, Vision

## Abstract

**Background:**

Pulleys have been reported as the functional origins of the rectus extraocular muscles (EOMs). However, biomechanical significance of pulleys on binocular vision has not been reported.

**Methods:**

Three eye movement models, i.e., non-pulley model, passive-pulley model, and active-pulley model, are used to simulate the horizontal movement of the eyes from the primary position to the left direction in the range of 1°–30°. The resultant forces of six EOMs along both orthogonal directions (i.e., the x-axis and y-axis defined in this paper) in the horizontal plane are calculated using the three models.

**Results:**

The resultant force along the y-axis of the left eye for non-pulley model are significantly larger than that of the other two pulley models. The difference of the force, between the left eye and the right eye in non-pulley model, is larger than those in the other two pulley models along x-axis and y-axis.

**Conclusion:**

The pulley models present more biomechanical advantage on the horizontally binocular vision than the non-pulley model. Combining with the previous imaging evidences of pulleys, the results show that pulley model coincides well with the real physiological conditions.

## Background

Connective tissue pulleys have been reported as the functional origins of the rectus extraocular muscles (EOMs) and can determine the effective pulling direction of each rectus [1, 2]. Most of the evidences for the existence of pulleys are from researches using various imaging technologies [3, 4]. The clinical application of connective tissue pulleys is gradually developed, such as pulley posterior fixation procedure [5, 6] and strabismus induced by pulley heterotopy [7]. Eye movement is controlled by six EOMs, i.e., lateral rectus (LR), medial rectus (MR), superior rectus (SR), inferior rectus (IR), superior oblique (SO) and inferior oblique (IO). Therefore, the forces of EOMs are responsible for eye movement [8]. Pulleys of EOMs may play an important role in making both eyes cooperate with each other, the biomechanical mechanism of which is nearly unknown. Because of the difficulties of the human EOMs anatomy, the ethical and moral restrictions and inevitably invasive characteristics, experiments of EOMs were recently performed on animals [9–11]. The modeling method is used to study the biomechanical significance of pulleys on binocular vision during the horizontal eye movement.

Many studies on the modeling of pulley have been reported. In tertiary gaze positions, each of the four rectus pulleys translated anteriorly and posteriorly with EOM relaxation and contraction, respectively. And the translation predicted by the active-pulley hypothesis was 100 times greater than that by a passive model [12]. The level of muscular activation also has been estimated using a model established with the concept of a pulley [13]. Recently, the eye movement model containing an immobile pulley, was applied to the study of strabismus [14]. Moreover, pulleys were contained in physically-based modeling and EOM simulation [15]. However, few studies have reported the effect of pulley on the force of EOMs and the vision.

Three eye movement models have been proposed to simulate the human eye movement: non-pulley model (traditional model), passive-pulley model, and active-pulley model. In the non-pulley model [16], the EOMs are constituted by the key points: insertion point, tangency point, and origin point (the end of the EOM attaching to the bony orbit). Subsequently, the concept of passive pulley was proposed [4]. In the passive pulley model [4, 17], the EOMs slide freely through the pulley, which is elastically stabilized relative to the orbital wall. Demer et al. [18] proposed the active-pulley hypothesis. Miller [19] proposes that in the active-pulley model the axes of rotation of EOMs tilt half of the angle of eye rotation in the first gaze, and the Listing’s law is implemented in the secondary and the tertiary gaze [12, 18], which is the foundation of the present work. Many reviews [12, 19–22] have described the active behavior of pulleys. However, these researchers only emphasized that pulley is the functional origin of EOMs.

Miller and Shamaeva [17] reported that the non-pulley EOMs can reasonably simulate normal and abnormal binocular alignment [19]. In this work, three eye movement models are used to analyze the effect of pulley on forces of EOMs and the biomechanical significance of pulley on vision.

## Methods

**Fig. 1:**
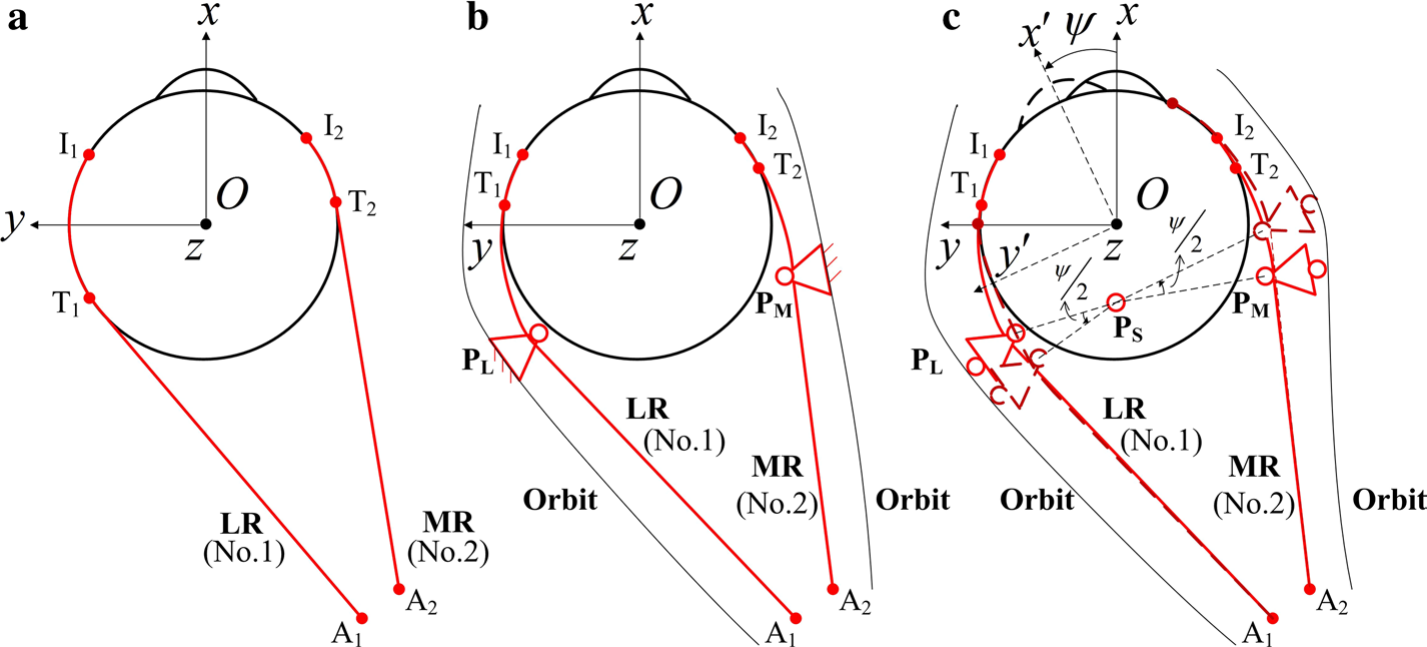
Schematics of three models of left eye. **a** Non-pulley model; **b** passive-pulley model; **c** active-pulley model (Revised from work by Demer et al. [20]). P_L_, P_M_, and P_S_ are the pulleys of LR, MR, and SR, respectively. The EOMs are plotted by the *red lines*. Not all the six EOMs are plotted. I_*i*_: the insertion point of the *i*th EOM; T_*i*_: the tangency point of the *i*th EOM; A_*i*_: the origin point of the *i*th EOM

The present study focuses on the effect of pulleys on the force of human EOMs. Three eye movement models (Fig. 1), i.e., non-pulley model (Fig. 1a), passive-pulley model (Fig. 1b), and active-pulley model (Fig. 1c), are used to simulate the horizontal eye movement from the primary position to the left direction in the range of 1°–30° within the normal physiological conditions [14].

**Fig. 2:**
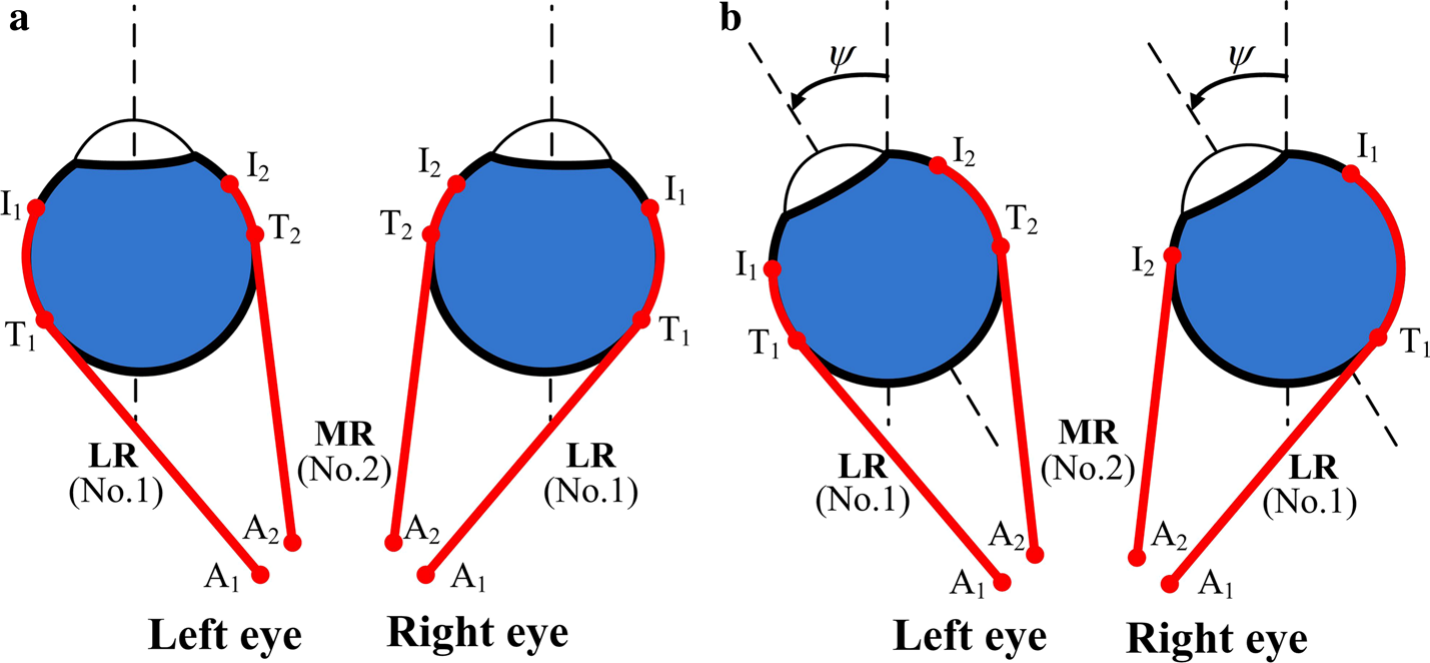
Schematic of horizontal movement of two eyes in non-pulley model. **a** The eyes are in the primary position; **b** the eyes rotate a angle ψ to the left direction. The EOMs are plotted by the *red lines*. Not all the six EOMs are plotted

The schematic of horizontal movement of two eyes in the non-pulley model is shown in Fig. 2. The eyeball, whose center is fixed, is set as a rigid sphere with a radius R = 12.43 mm and the EOMs are represented by strings which can contract actively [16, 23]. The geometry parameters of EOMs of the left eye are shown in Table 1 [1, 13, 16]. The corresponding y-coordinates of right eye are the negative values of those of left eye. The other coordinates of right eye are the same as those of the left eye.

**Table 1:** Geometrical parameters of extraocular muscles of left eye

	Origin point (mm) [18]			Insertion point (mm) [18]			Pulley point (mm) [1]			Area (mm^2^) [15]
	x	y	z	x	y	z	x	y	z	–
LR	−34.00	−13.00	0.60	6.50	10.08	0.00	−11.00	10.02	−0.50	16.73
MR	−30.00	−17.00	0.60	8.84	−9.65	0.00	−5.00	−14.40	−0.60	17.39
SR	−31.76	−16.00	3.60	7.63	0.00	10.48	−7.63	0.00	11.60	11.34
IR	−31.76	−16.00	−2.40	8.02	0.00	−10.24	−7.63	−4.50	−13.00	15.85
SO	8.24	−15.27	12.25	−4.41	2.90	11.05	–	–	–	19.34
IO	11.34	−11.10	−15.46	−7.18	8.70	0.00	–	–	–	19.83

In the non-pulley model, the pulleys are not included. In the passive-pulley model, the pulleys of the four rectus EOMs are considered, and all the pulleys are immobile. In the active-pulley model, the pulleys of the four rectus EOMs are included. In the horizontal eye movement, to simply the active-pulley model, only the pulleys of horizontal recti (LR and MR) are mobile [18], while the pulleys of the SR and IR are immobile.

### Translation relationships

The Oxyz is defined as stationary coordinate system, and the Ox′y′z is defined as body axes system of eyeball (Fig. 1c). The (x′, y′, z) is the coordinate of a point on eyeball in body axes system Ox′y′z, and the coordinate of this point in stationary coordinate system Oxyz is set as (x, y, z). The (x_Ii_′, y_Ii_′, z_Ii_) is the coordinate of the insertion point of the *i*th EOM on the eyeball in body axes system Ox′y′z, and the (x_Ii_, y_Ii_, z_Ii_) is the corresponding coordinate in stationary coordinate system Oxyz. The translation relationship between the coordinates (x_Ii_′, y_Ii_′, z_Ii_) and (x_Ii_, y_Ii_, z_Ii_) is shown by Eq. (1). The Eq. (2) is the corresponding translation relationship of the mobile pulley coordinates [24].

1 $$\left( \begin{aligned} x_{{{\text{I}}i}} \hfill \\ y_{{{\text{I}}i}} \hfill \\ z_{{{\text{I}}i}} \hfill \\ \end{aligned} \right) = \left[ {\begin{array}{*{20}c} {\cos \psi } & { - \sin \psi } & 0 \\ {\sin \psi } & {\cos \psi } & 0 \\ 0 & 0 & 1 \\ \end{array} } \right]\left( \begin{aligned} x^{\prime}_{{{\text{I}}i}} \hfill \\ y^{\prime}_{{{\text{I}}i}} \hfill \\ z_{{{\text{I}}i}} \hfill \\ \end{aligned} \right) .$$

2 $$\left( \begin{aligned} x_{\text{ph}} - x_{\text{pv}} \hfill \\ y_{\text{ph}} - y_{\text{pv}} \hfill \\ z_{\text{ph}} - z_{\text{pv}} \hfill \\ \end{aligned} \right) = \left[ {\begin{array}{*{20}c} {\cos \left( {{\psi \mathord{\left/ {\vphantom {\psi 2}} \right. \kern-0pt} 2}} \right)} & { - \sin \left( {{\psi \mathord{\left/ {\vphantom {\psi 2}} \right. \kern-0pt} 2}} \right)} & 0 \\ {\sin \left( {{\psi \mathord{\left/ {\vphantom {\psi 2}} \right. \kern-0pt} 2}} \right)} & {\cos \left( {{\psi \mathord{\left/ {\vphantom {\psi 2}} \right. \kern-0pt} 2}} \right)} & 0 \\ 0 & 0 & 1 \\ \end{array} } \right]\left( \begin{aligned} x^{\prime}_{\text{ph}} - x_{\text{pv}} \hfill \\ y^{\prime}_{\text{ph}} - y_{\text{pv}} \hfill \\ z_{\text{ph}} - z_{\text{pv}} \hfill \\ \end{aligned} \right) .$$

In Eq. (2), the subscript *ph* represents the pulley of horizontal recti, and the subscript *pv* represents the pulley of SR.

### Tangency point of EOMs

The coordinates T_i_ (x_Ti_, y_Ti_, z_Ti_) of the tangency point of the *i*th EOM in non-pulley model can be calculated using Eq. (3), and those of the other two pulley models can be calculated using Eq. (4). In the set of Eq. (3) or (4), the first equation can ensure that the tangency point is in the spherical surface of eyeball, the second equation means that the EOM is tangent to the spherical surface of eyeball, and the third equation represents that the tangency point is in the plane OA_*i*_I_*i*_ (Fig. 1). The value of the subscript *i* ranges from 1 to 6, which represents the LR, MR, SR, IR, SO, and IO muscles, respectively.

3 $$\left\{ {\begin{array}{*{20}l} {x_{{{\text{T}}i}}^{2} + y_{{{\text{T}}i}}^{2} + z_{{{\text{T}}i}}^{2} = {\text{R}}^{2} } \\ {x_{{{\text{T}}i}} \cdot x_{{{\text{A}}i}} + y_{{{\text{T}}i}} \cdot y_{{{\text{A}}i}} + z_{{{\text{T}}i}} \cdot z_{{{\text{A}}i}} = {\text{R}}^{2} } \\ {\left( {y_{{{\text{A}}i}} z_{{{\text{I}}i}} - z_{{{\text{A}}i}} y_{{{\text{I}}i}} } \right)\cdot x_{{{\text{T}}i}} + \left( {z_{{{\text{A}}i}} x_{{{\text{I}}i}} - x_{{{\text{A}}i}} z_{{{\text{I}}i}} } \right)\cdot y_{{{\text{T}}i}} + \left( {x_{{{\text{A}}i}} y_{{{\text{I}}i}} - y_{{{\text{A}}i}} x_{{{\text{I}}i}} } \right)\cdot z_{{{\text{T}}i}} = 0} \\ \end{array} } \right.$$

4 $$\left\{ {\begin{array}{*{20}l} {x_{{{\text{T}}i}}^{2} + y_{{{\text{T}}i}}^{2} + z_{{{\text{T}}i}}^{2} = {\text{R}}^{2} } \\ {x_{{{\text{T}}i}} \cdot x_{{{\text{P}}i}} + y_{{{\text{T}}i}} \cdot y_{{{\text{P}}i}} + z_{{{\text{T}}i}} \cdot z_{{{\text{P}}i}} = {\text{R}}^{2} } \\ {\left( {y_{{{\text{P}}i}} z_{{{\text{I}}i}} - z_{{{\text{P}}i}} y_{{{\text{I}}i}} } \right)\cdot x_{{{\text{T}}i}} + \left( {z_{{{\text{P}}i}} x_{{{\text{I}}i}} - x_{{{\text{P}}i}} z_{{{\text{I}}i}} } \right)\cdot y_{{{\text{T}}i}} + \left( {x_{{{\text{P}}i}} y_{{{\text{I}}i}} - y_{{{\text{P}}i}} x_{{{\text{I}}i}} } \right)\cdot z_{{{\text{T}}i}} = 0{\text{ }}} \hfill \\ \end{array} } \right.$$

where subscripts T_*i*_, A_*i*_, I_*i*_, and P_*i*_ represent tangency point, origin point, insertion point and pulley of the *i*th EOM.

### Forces of EOMs

When the eyeball is in equilibrium, the governing equation is

5 $$\sum\limits_{i = 1}^{6} {{\mathbf{r}}_{i} \times {\mathbf{F}}_{i} + {\mathbf{M}}_{\text{t}} = 0} ,$$

where the **r**_*i*_ and **F**_*i*_ are the radius vector and force vector of the *i*th EOM, and the resisting moment of the other orbital tissues is **M**_t_ = −*ψ*K_t_R^2^ ***k***, in which restraining stiffness K_t_ = 1.245 mN/(°) [25], and the ***k*** is the unit vector along z-axis. The magnitude of the **F**_*i*_ of the *i*th EOM is F_*i*_ = F_a*i*_ + F_p*i*_ (*i* = 1, 2, …, 6) [26], where the subscripts *a* and *p* denote the active force and passive force, respectively. F_p*i*_ can be described as [27].

6 $${\text{F}}_{{{\text{p}}i}} = 1.02{\text{c}}_{i} \exp \left( {{{\Delta {\text{L}}_{i} } \mathord{\left/ {\vphantom {{\Delta {\text{L}}_{i} } {3.15}}} \right. \kern-0pt} {3.15}}} \right) ,$$

where c_*i*_ = 2A_*i*_/(A_1_ + A_2_), A_*i*_ and ΔL_*i*_ are the cross-section and elongation of the *i*th EOMs, respectively.

When the left eye rotates to the left, the MR, SR, and IR are all elongate passively and they are antagonists, and the other three EOMs are agonists. We assume that the active force F_a*i*_ of the antagonist is zero, the total forces of MR, SR, and IR can be calculated by Eq. (6). In this case, the total forces of the other three EOMs can be calculated by Eq. (5). Thus, the resultant forces of the six EOMs along the x- and y-axes can be obtained. The calculating process of the forces of EOMs for right eye is similar to that of the left eye.

## Results

When the eye looks straight forward with the head fixed and upright (primary position), the anatomical locations of the EOMs of left eye are symmetrical to those of the right eye (Fig. 2a). According to Hering’s law [28], the right eye will rotate the same angle along the same direction as the left eye rotating an angle. In this case, the anatomical locations of the EOMs between the left eye and right one are no longer symmetrical (Fig. 2b), and the forces of the EOMs of the left eye and right one may be different.

### Comparison of resultant force along y-axis in three models

**Fig. 3:**
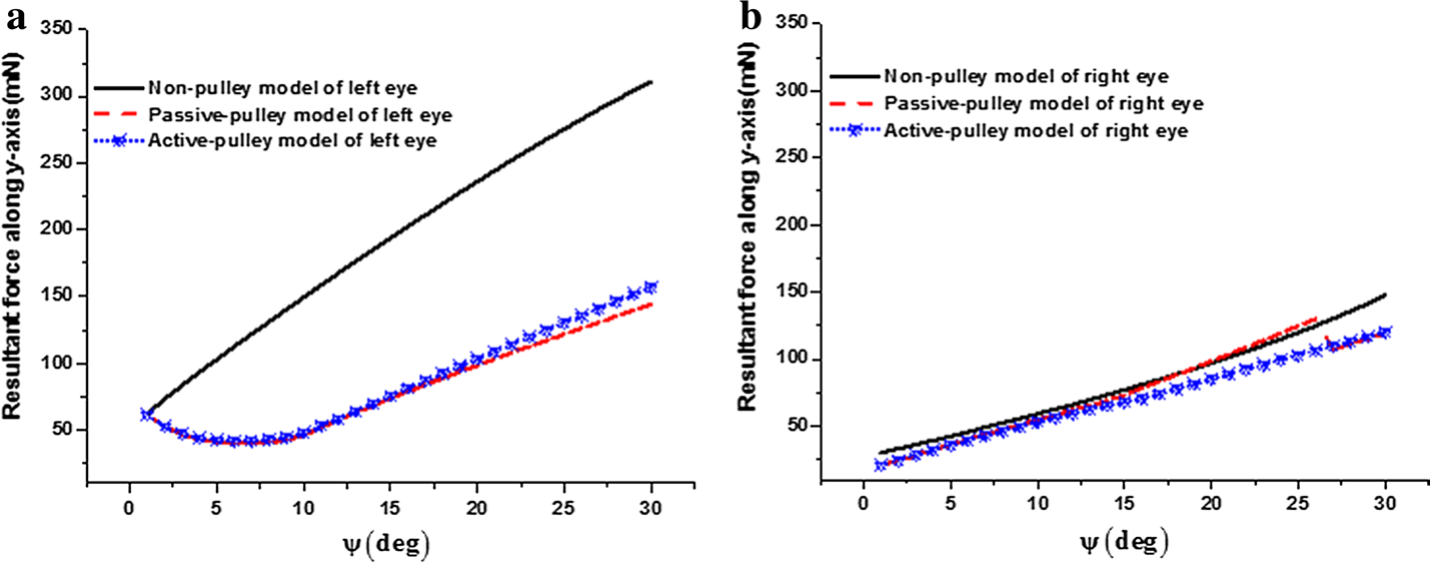
Comparison of resultant force along y-axis in three models. **a** Left eye; **b** right eye

Comparisons of resultant force along y-axis in the three models of the left and right eyes are shown in Fig. 3. For the left eye (Fig. 3a), the resultant force along y-axis linearly increases from 62.58 to 311.08 mN with the eye rotating left from 1° to 30° in non-pulley model. Meanwhile, in the passive-pulley model, the resultant force nonlinearly decreases from 61.33 to 40.15 mN with the eye rotating from 1° to 7° and increases to 144.40 mN with the eye rotating to 30°. Similarly, in the active-pulley model, the resultant force nonlinearly decreases from 61.39 to 41.92 mN with the eye rotating from 1° to 7° and increases to 156.86 mN with the eye rotating to 30°. The resultant forces along the y-axis are nearly the same between passive-pulley and active-pulley models. For the non-pulley model of left eye, the resultant force along y-axis is nearly 160 mN larger than those of the other two pulley models with the eye rotating 30° to the left. For the right eye (Fig. 3b), the resultant forces along y-axis are nearly close in three models as the eye rotates to the left.

### Comparison of resultant forces along x-axis between two eyes in three models

**Fig. 4:**
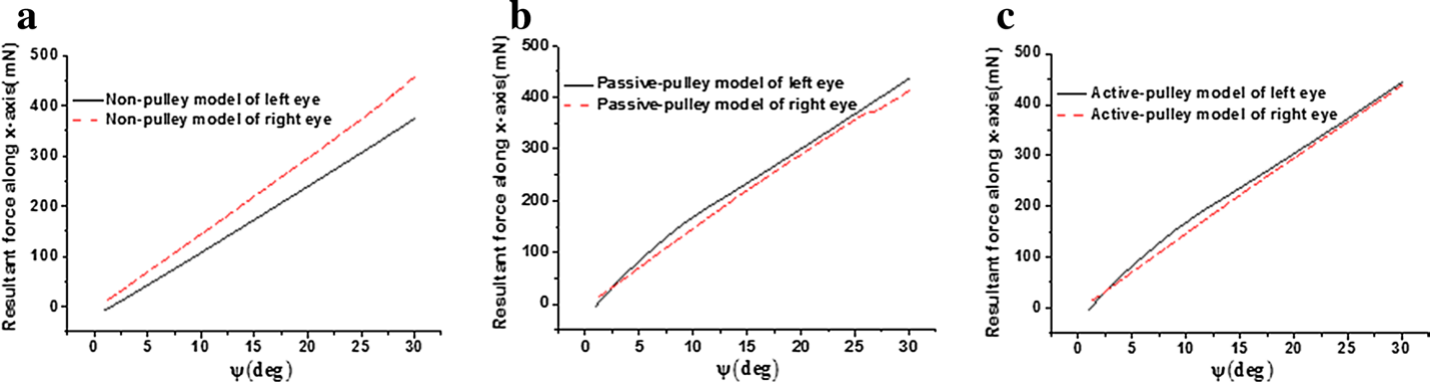
Comparison of resultant forces along x-axis between two eyes in three models. **a** Non-pulley model; **b** passive-pulley model; **c** active-pulley model. The ψ denotes the angle of the eye rotating to the left

Comparisons of resultant forces along x-axis between two eyes in the three models are shown in Fig. 4. For the non-pulley model (Fig. 4a), the resultant forces along the x-axis of the two eyes increase linearly. This force reaches its maximum value of 457.44 mN with the right eye rotating 30° to the left, whereas that of the left eye reaches to 373.66 mN with the eye rotating 30° to the left. The difference of the forces between two eyes becomes larger with the eye rotating from 1° to 30°, and it reaches up to nearly 83.78 mN with the eyes rotating to 30°. Moreover, for the other two pulley models (Fig. 4b, c), the resultant forces along the x-axis are nearly the same between two eyes.

### Comparison of resultant forces along y-axis between two eyes in three models

**Fig. 5:**
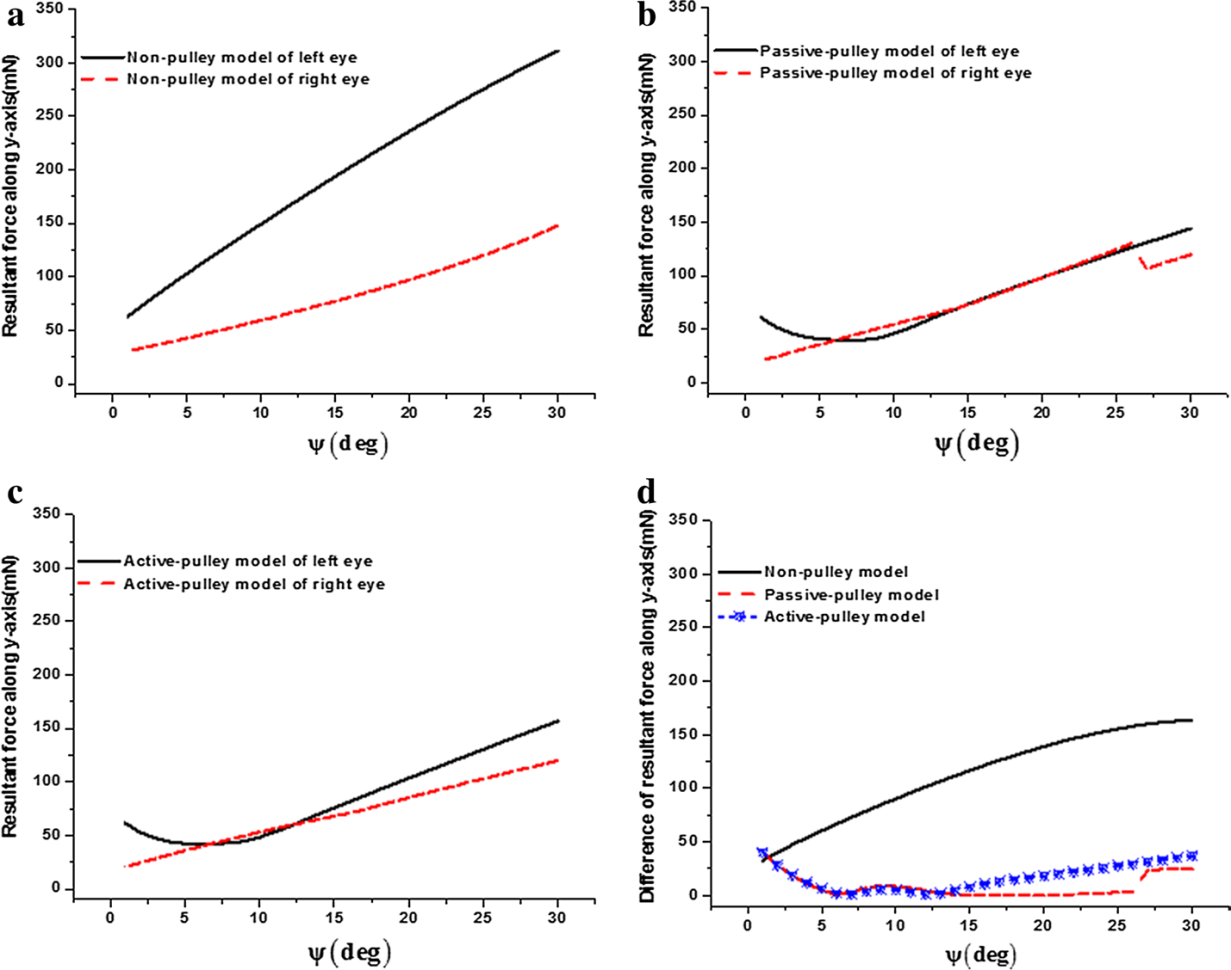
Comparison of resultant forces along y-axis between two eyes in three models. **a** Non-pulley model; **b** passive-pulley model; **c** active-pulley model; **d** difference of resultant force along y-axis between two eyes in three models. The ψ denotes the angle of the eye rotating to the left

Comparisons of the resultant forces along the y-axis between both eyes simulated by the three models are shown in Fig. 5. For the non-pulley model, the resultant forces of both eyes along the y-axis trend to linearly increase (Fig. 5a). The largest value of the right eye is 147.84 mN on the 30° position of the left side, and the value of the left eye is 311.08 mN in the same position. However, resultant forces along the y-axis in the passive-pulley model and the active-pulley model are less than that in non-pulley model (Fig. 5b–d). The curves shown in Fig. 5d reveal the differences of the resultant force along the y-axis between two eyes simulated by the three models; i.e., when the eyes horizontally move in the range of 1°–30°, this difference in non-pulley model is larger than those in the two pulley models. Whereas the differences in the two pulley models are not significantly different.

## Discussion

The forces of the EOMs are calculated in three models. For simplification, the center of eyeball is fixed in the modeling. In fact, the eye center can move in a small range with the eyeball moving [16]. The center of eyeball may be moved by the resultant forces of EOMs; meanwhile the eyeball will be deformed by those forces. Compared with the two pulley models, the greater resultant force along the y-axis obtained by the non-pulley model may induce larger deformation of the left eye (Fig. 3a). This may lead to the deformation of the cornea, crystalline lens, and fovea of the left eye, and then the corresponding y-coordinates may change more largely in the non-pulley model than in the two pulley models. Meanwhile, in the non-pulley model, the light path of the object differs from the physiological condition. Therefore, the deformation of the left eye may affect the judgment to the position of an object if pulleys do not exist. This means that the existence of pulleys reduces the disadvantage of non-pulley situation to the horizontal vision, which confirms the previous conclusion of the advantage of pulley [13–15, 19, 29].

The difference of the resultant force of EOMs along the x-axis can induce different translations along this direction between both eyes. The resultant forces along x-axis direct to the back of orbit. The differences of these forces between two eyes (Fig. 4a) will result in the different deformations and x-coordinates of the centers between two eyes. Thus, the x-coordinates of the cornea and crystalline lens between the two eyes may become different. Meanwhile, the light path of the object may be influenced. Therefore, judgment to position and distance of the object is affected.

**Fig. 6:**
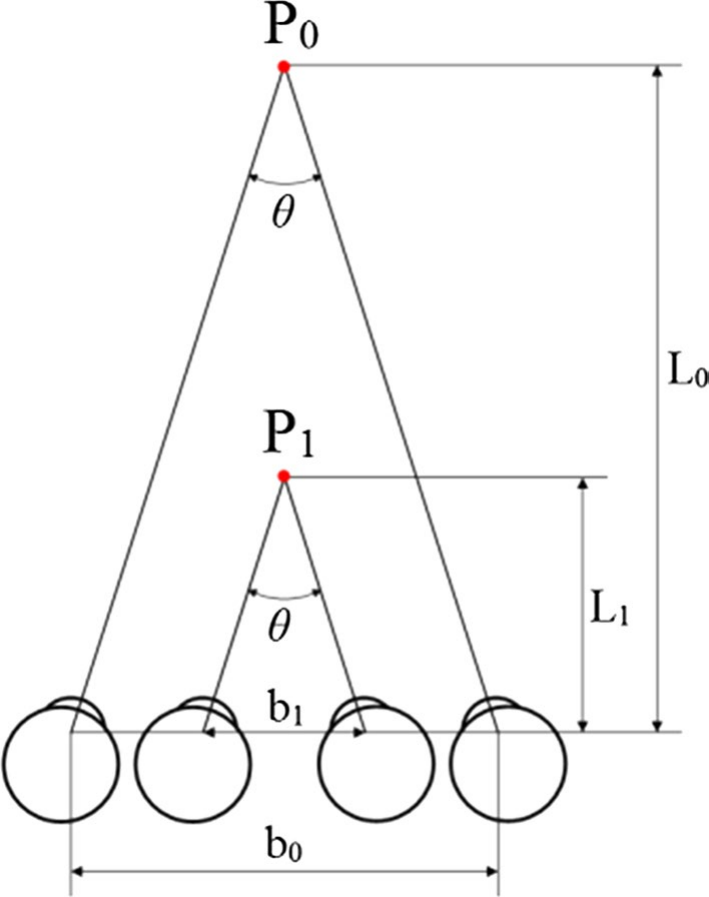
Comparison of binocular visions between two different interocular distances b_0_ and b_1_. The fixation points P_0_ and P_1_ denote two different objects; b_0_ and b_1_ are the corresponding interocular distances, and b_0_ > b_1_; L_0_ and L_1_ are the corresponding distances from the object points P_0_ and P_1_ to the eye, respectively; angle *θ* is the corresponding binocular subtense angle of the object, and *θ* = b_0_/L_0_

The direction of the resultant force along the y-axis of the left eye is negative, and that of the right eye is positive. In addition, the differences of the forces (Fig. 5d) between two eyes can result in the different deformations and the smaller center distances between two eyes relative to their primary distance. As a result, judgment to position and distance of the object is affected. The different deformations may change the relative distances of the cornea, crystalline lens, and fovea between the two eyes. The light path may be changed and the judgment to position and distance of the object is eventually affected. Moreover, when eyes observe an object, binocular subtense angle vary with the different distances of the object. According to the different subtense angles, the distance of the object can be identified. When the center distance of two eyes changes, objects at different distances may result in the same binocular subtense angle. Comparison of binocular visions between two different interocular distances b_0_ and b_1_ is shown in Fig. 6; b_0_ denotes the normal interocular distance, and b_1_ represents the decreased interocular distance. When the binocular subtense angle is *θ*, the object P_0_ is judged at the distance of L_0_. However, when the interocular distance changes from b_0_ to b_1_, the object P_1_ at the distance of L_1_ is misjudged at the distance of L_0_ because its binocular subtense angle remains *θ.* In this case, the object near the eye is judged as a far one. In consequence, the variation of interocular distance may lead to the wrong judgment of distance of the object.

Moreover, in the non-pulley model, the differences of the resultant force along the x- or y-axes between two eyes increase with horizontal eye movement (Figs. 4a, 5a). Therefore, the center coordinates of two eyes vary continuously with eye movement, which leads that the judgment to the distance of object changes continuously.

The pulley models coincide well with the real physiological conditions. Thus the models can direct the clinical ophthalmology properly. In the normal binocular vision, the optical axis of the left eye is parallel to that of the right eye. When the strabismus occurs, these two optical axes will not be parallel to each other. The horizontal strabismus can be treated by enhancing the strength of some EOM (EOM resection) or weakening the strength of some EOM (EOM recession) by surgical operation. However, the determination of the surgical amounts of the EOMs relies on the experience of the clinician during surgery [30, 31], and there may be some error. The pulley models, because they are close to the actual physical situation, can be used to determine the surgical amounts of the EOMs in strabismus and provide a theoretical reference to the clinician for the treatment of many other eye movement disorders.

## Conclusion

The resultant forces along the x- and y-axes in three eye movement models are obtained in this paper. The calculation results show that the resultant forces along the y-axis of the left eye for non-pulley model are significantly different from those of the other two pulley models. Compared with the other two pulley models, for the non-pulley model the resultant forces along the x- and y-axes distinctly differ between two eyes. The translation and deformation of eyeball are less in pulley model than those in non-pulley model. Therefore, the pulley model presents more biomechanical advantage on the horizontally binocular vision than the non-pulley model, and the existence of pulley is biomechanically significant. The pulley models can be used to provide a theoretical reference to the clinician for the treatment of eye movement disorders.
